# Nanoparticle-mediated synergistic chemoimmunotherapy for tailoring cancer therapy: recent advances and perspectives

**DOI:** 10.1186/s12951-021-00861-0

**Published:** 2021-04-17

**Authors:** Rafieh Bagherifar, Seyed Hossein Kiaie, Zahra Hatami, Armin Ahmadi, Abdolvahid Sadeghnejad, Behzad Baradaran, Reza Jafari, Yousef Javadzadeh

**Affiliations:** 1 Student Research Committee, Tabriz University of Medical Sciences, Tabriz, Iran; 2 Department of Pharmaceutics, Faculty of Pharmacy, Tabriz University of Medical Sciences, Tabriz, Iran; 3 Nano Drug Delivery Research Center, Kermanshah University of Medical Sciences, Kermanshah, Iran; 4 Department of Immunology, Faculty of Medical Sciences, Tarbiat Modares University, Tehran, Iran; 5 Department of Chemical & Materials Engineering, The University of Alabama in Huntsville, Huntsville, AL 35899 USA; 6 Cancer Research Center, Semnan University of Medical Sciences, Semnan, Iran; 7 Immunology Research Center, Tabriz University of Medical Sciences, Tabriz, Iran; 8 Solid Tumor Research Center, Cellular and Molecular Medicine Institute, Urmia University of Medical Sciences, Shafa St, Ershad Blvd., P.O. BoX: 1138, 57147 Urmia, Iran; 9 Department of Immunology and Genetics, School of Medicine, Urmia University of Medical Sciences, Urmia, Iran; 10 Biotechnology Research Center, and Faculty of Pharmacy, Tabriz University of Medical Science, 5166-15731 Tabriz, Iran

**Keywords:** Chemoimmunotherapy, Chemotherapy, Immunotherapy, Nanoparticles, Monoclonal antibody, Cytokines

## Abstract

Nowadays, a potent challenge in cancer treatment is considered the lack of efficacious strategy, which has not been able to significantly reduce mortality. Chemoimmunotherapy (CIT) as a promising approach in both for the first-line and relapsed therapy demonstrated particular benefit from two key gating strategies, including chemotherapy and immunotherapy to cancer therapy; therefore, the discernment of their participation and role of potential synergies in CIT approach is determinant. In this study, in addition to balancing the pros and cons of CIT with the challenges of each of two main strategies, the recent advances in the cancer CIT have been discussed. Additionally, immunotherapeutic strategies and the immunomodulation effect induced by chemotherapy, which boosts CIT have been brought up. Finally, harnessing and development of the nanoparticles, which mediated CIT have expatiated in detail.
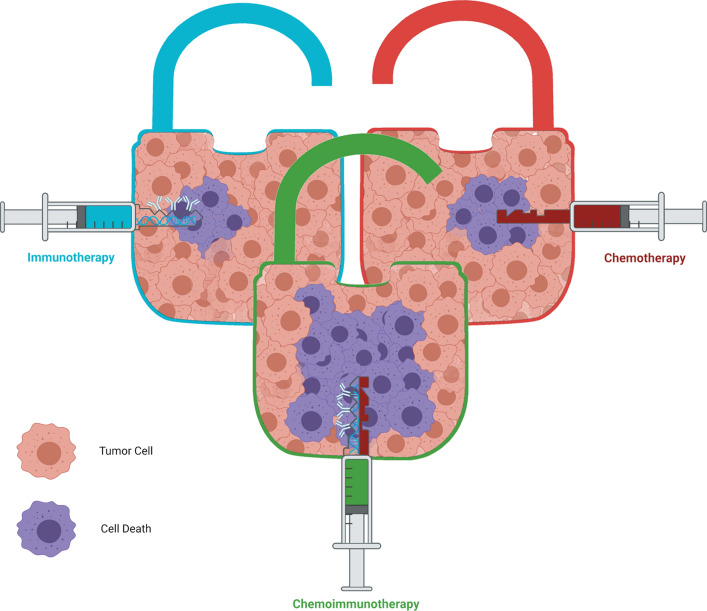

## Introduction

Despite advances in promoting knowledge about cancer initiation and progress, utilization of primary, adjuvant, and palliative treatment approaches, and the development of innovative therapies, overall cancer patient survival rates have slightly improved. Cancer treatments based on palliative treatment approaches alone, such as chemotherapy, immunotherapy, and radiotherapy could not display considerable efficacy [1–3]. Therefore, the expansion of new strategies to control survival and death in cancer therapy is necessary [4–6]. Over the past decades, chemotherapy used drugs with anti-cancer activity that inhibit proliferation of cancer cells, divided in an uncontrolled manner. The struggle to discover effective chemotherapeutic drugs came back to the beginning of the twentieth century. For the first time in 1948, chemodrug agents improved acute lymphoblastic leukemia (ALL) in children. In the 1950s, Eli Lilly and Company presented the benefit of plant alkaloids (from Vinca rosea) for ALL patients, and in 1957, 5-fluorouracil was discovered with broad-range activity against many solid tumors [7, 8]. Chemotherapy, depending on the cancer type, has been used to prevent cancer relapse, inhibit metastasis, accelerate the tumor shrinkage, and reduce the tumor pressure effect. The most common action mechanisms include alkylating agents, antimetabolites, mitotic spindle inhibitors, and topoisomerase inhibitors [9, 10]. Chemotherapy not only impacts tumor cells but also overwhelms healthy cells; therefore, these adverse effects could also affect normal cell functions. According to the unspecified distribution and multidrug resistance (MDR) of chemotherapeutic drugs, it could cause some disadvantages such as rapid clearance and poor pharmacokinetics (PK), as well as numerous adverse effects [11, 12]. The adverse effects of chemotherapy might be determined by different factors such as drug (type and dose) and cancer ( class and location), as well as the general health status of patients [13, 14]. Likewise, the most common adverse effects of chemotherapy include opportunistic infectious diseases, fatigue, hair loss, diarrhea, nausea and vomiting, anemia, easy bruising and bleeding, and pain such as headaches and stomach and muscle pains [15].

Lately, cancer immunotherapy, which improves the anti-tumor immune responses through stimulation or suppression of the immune system components and activity, displays encouraging results in cancer treatment [16, 17]. Immunotherapy currently has been a tremendous interest in developing a broad spectrum of cancer therapy from the cold tumor, such as cervical and pancreatic cancers to the hot tumor, such as lung cancer and melanoma [18, 19].

The first scientific attempt at modulating the function of the immune system to treat cancer by Fehleisen and Busch was occurred in 1974 [20]. William B. Coley announced the second significant effort to utilize the immune system to treat bone cancer in 1891 [21, 22]. Coley’s principles were established and planned under a clinical trial by Old et al. in 1959 [23]. During this same decade, the concept of immunosurveillance was established by Thomas [24] and Burnet [25, 26]. In comparison to traditional therapies, immunotherapy by using the immune system to fight tumor cells and due to selectivity and long-lasting effects, demonstrates overall survival benefit in preclinical studies and less toxicity on healthy cells leading to the reduction of the adverse effects which followed by traditional therapies [27, 28]. However, cancer cells evade the immune system by creating a suppressive microenvironment using various strategies such as expressing inhibitory molecules or recruiting cells to secrete suppressive compounds, leading to a decrease in the effectiveness of immunotherapeutic approaches. Therefore, modulation of the immune-suppressive tumor microenvironment (TME) is a pivotal player in cancer immunotherapy, which inhibits the immune system's suppressive factors and promotes the function of the components of the immune system [29–32]. In contrast, the most common obstacles in cancer immunotherapy include unpredictable efficacy due to variability in target mutations, unknown cancer biomarkers and pathways, tumor heterogeneity, immunosuppression and biomarker identification such as technical limitation for recognition of predictive genetic mutations, and cost [6, 33]. Despite successful cancer treatment achievements by using immunotherapy or chemotherapy alone, the limitations hindered the harnessing, development, and administration of each of the immunotherapy or chemotherapy approaches alone in cancer therapy.

**Fig. 1:**
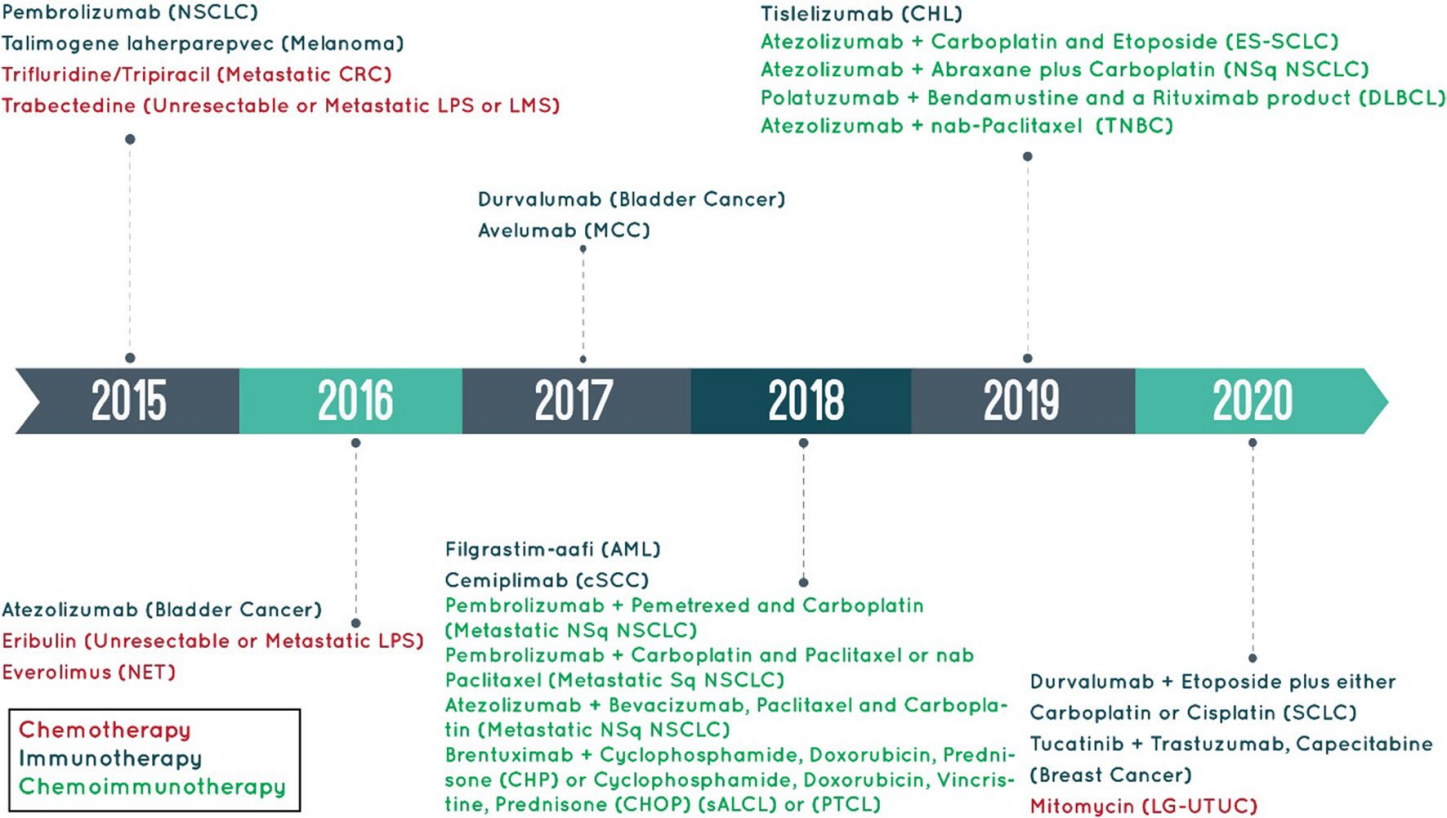
A timeline for FDA-approved products for chemotherapy, immunotherapy and chemoimmunotherapy from 2015 to 2020. *SCLC* small cell lung cancer, *NSCLC* non-small cell lung cancer, *LMS* leiomyosarcoma, *LPS* liposarcoma, *CRC* colorectal cancer, *NET* neuroendocrine tumors, *MCC* merkel cell carcinoma, *AML* acute myeloid leukemia, *cSCC* cutaneous squamous cell carcinoma, *NSq NSCLC* non-squamous non-small cell lung cancer, *sALCL* systemic anaplastic large cell lymphoma, *PTCL* peripheral T cell lymphomas, *CHL* classical hodgkin lymphoma, *ES-SCLC* extensive-stage small cell lung cancer, *TNBC* triple-negative breast cancer, *DLBCL* diffuse large B cell lymphoma, *LG-UTUC* low-grade upper tract urothelial cancer

Chemoimmunotherapy (CIT) is able to combine and use both traditional chemotherapy and current immunotherapy approaches to inhibit tumor progression, metastasis and recurrence even if it is not possible to obtain a cure or relieve the symptom in palliative care [34, 35]. Chemotherapy drug firstly kills the tumor cells and generates cross-presented tumor antigens, making the tumor as a source of tumor antigens. Afterwards, the simultaneous or sequential administration of immunotherapeutic agents leads to stimulation of the tumor antigens and immunostimulants to create a potent anti-tumor immune response [36]. Moreover, immunotherapy could overcome the limitations arising from low specificity and high drug resistance of chemotherapy agents while enhancing sensibility of tumor cells to chemotherapy agents [37, 38]. Therefore, this combination system could increase therapeutic effectiveness through synergistic effects. Moreover, it has been demonstrated that some chemotherapy drugs alone induce immunomodulation effects through immunogenic cell death (ICD), sensitizing tumor cells to immune assault, and elimination of immunosuppressive cells in the TME. These drugs show the potential for CIT as single agent or in combination with other chemotherapy or immunotherapy drugs [39–41]. CIT has been used in pre-clinical studies or even in clinical trials and in different types of cancers, especially melanoma, breast cancer, hepatic tumor, prostate cancer, and lung cancer [42]. A study analysis of Food and Drug Administration (FDA)-approved products for chemotherapy, immunotherapy and CIT approaches highlighted harnessing and development of CIT for the prospects of more clinical use in cancer treatment. A pivotal events timeline for FDA-approved chemotherapy, immunotherapy, and CIT products with year from 2015 to 2020 are pointed out in Fig. 1. This review aims to discuss a compelling role of CIT as potential synergies of tailored cancer, which begins with a discussion of immunotherapeutic strategies and chemotherapy with immunogenic effect which boost CIT and interplay of chemotherapy and overlap between innate and adaptive immunity. Then, potential of nanoparticles (NPs)-assisted CIT illustrates the promising way in which prospect of the CIT researches will be considered. To that end, carrier-mediated CIT with the combination of chemotherapy drugs and immunotherapeutic agents, including cytokines, immune adjuvants, monoclonal antibodies (mAbs), and other immunotherapy agents for synergistic cancer therapy is explored in detail.

## Immunotherapeutic strategies to boost chemoimmunotherapy

Immunotherapy is classified into two types, active and passive, based on the patients' immune system status and the mechanism of immunotherapeutic agents. Passive immunotherapy, which utilizes immunotherapeutic agents like cytokines, tumor-specific mAbs, adoptive cell transfer therapy (ACT), and immune adjuvants optimizes the host’s immune system to fight tumor cells efficiently, rather than inducing cancer cell death. In comparison to active immunotherapy, which creates long-lasting immunity, passive immunotherapy requires repeated administration [43, 44]. mAbs are proteins that engineered to bind to tumor-specific antigens and can be used alone or conjugated to specific medications, toxins, or radioactive agents and carry them to cancer cells [45]. ACT, also known as cellular immunotherapy, is the most effective immunotherapeutic approach, which involves isolating a cancer patient’s tumor-specific lymphocytes, ex vivo modification, activation and expansion, and subsequently, their reinfusion to the patient [46]. Three forms of ACT, including tumor-infiltrating T lymphocyte (TIL), chimeric antigen receptor (CAR) T cell, and engineered T cell receptor (TCR), have been developed for cancer therapy [47]. In CAR T cell- and TCR therapy, patient-derived T lymphocytes are engineered ex vivo to express artificial T cell receptors or equipped with synthetic T cell receptors, respectively. As a result, T lymphocytes gain the ability to target cancer cells [48, 49]. Unlike TIL and TCR, CARs can bind to tumor cells in an MHC-independent fashion [50]. Cytokines are a broad class of small soluble proteins secreted by certain cells like macrophages (MQs), T cells, B cells, and mast cells. They act as a mediator of cell communication, cause immune cells growth and differentiation, and regulate inflammatory or anti-inflammatory responses in various cell types [51]*.* Pro-inflammatory cytokines (PICs) in the initial steps of tumorigenesis display anti-tumor activity by improving antigen priming, stimulating immune effector cells, and increasing the number and cytotoxic activity of immune cells in the TME [52, 53]*.* As such, some cytokines can kill tumor cells either directly through providing anti-proliferative and pro-apoptotic tumor signals or indirectly by activating cytotoxic immune cells. Moreover, cytokines in combination with mAbs, which inhibit immune checkpoint (ICP) molecules such as programmed cell death ligand-1 (PD-L1) and programmed cell death protein-1 (PD-1), have been used in several clinical trials [54]*.* On the other hand, some cytokines like interleukin (IL)-10 and transforming growth factor-β (TGF-β), which are released from cancer cells and the TME cells, could promote tumorigenesis and suppress the immune system. In this case, different strategies including antagonistic antibodies, polypeptides, cytokine traps, and small molecules that inhibit cytokine receptor signal transduction are used to neutralize the immunosuppressive activity of cytokines [52]. To date, interferon-α (IFN-α) and IL-2 have received FDA approval for the treatment of several cancers as monotherapy [55, 56]. Adjuvant is a molecule that potentiates the innate immune responses through activation of pathogen recognition receptors (PRRs) like NOD-like receptors (NLRs) as well as toll like receptors (TLRs) by their agonists. Subsequently, this activation leads to the production of cytokine and chemokine, which in turn promote the activation and maturation of antigen-presenting cells (APCs) [57]. Lipopolysaccharide (LPS), cytosine-phosphate-guanosine oligodeoxynucleotides (CpG-ODN), agonists of stimulator of interferon genes (STING), and polyinosinic-polycytidylic acid (poly I:C) are commonly used adjuvants in cancer immunotherapy [58]. Thus, adjuvants have attracted more attention as a crucial component of cancer vaccines. Combining the adjuvants with tumor-specific antigens in different types of effective cancer vaccines has been developed in cancer immunotherapy. To that end, these adjuvant vaccines have been internalized into APCs using NPs that could enhance immunity against cancer [59].

On the other hand, active immunotherapy approaches cause in vivo activation of host’s immune system through stimulation of effector cells functions and induce cancer cell death through immune checkpoint inhibitors (ICIs), oncolytic viruses, and different types of anti-cancer vaccines such as peptide vaccines, whole-cell vaccines, and dendritic cell (DC)-based vaccines [60]. Moreover, the TME is usually immunosuppressive in various cancers. Thus, tumor immunotherapy's primary strategy is to disrupt immunosuppressed microenvironment while inducing an effective T cell response against tumor epitopes and providing a stable immunological memory against a wide repertoire of cancer epitopes [61]. Cancer cells do not express danger signals at the early stage, so the immune system cannot respond to tumor antigens. The DC vaccine stimulates anti-tumor immune responses in cancer patients to correct this failure by producing efficient antigen-specific T cells. The combination of DC vaccine with other immunotherapy agents is used in several clinical studies. For instance, the combination of DC vaccine with mAbs (anti-programmed cell death protein-1 (PD-1) and anti-CTLA-4), or other ICIs has been studied [62]. In another study, blockade of programmed cell death ligand-1 (PD-L1) expression on DCs, which enhanced T cell priming potential of DC vaccine has been indicated [63, 64]. Immune checkpoint (ICP) molecules, including CTLA-4 and PD-1/PD-L1 impede T cells' anti-tumor activity through the transmission of inhibitory signals leading to downregulation in immune responses [65, 66]. ICIs are mAbs that target ICP molecules and activate anti-tumor immunity with different types of malignancies such as prostate and pancreatic cancers, metastatic melanoma, renal cell carcinoma (RCC) and non-small cell lung cancer (NSCLC) [67–71]. Due to the high expression of PD-L1 in most tumor cells, cytokine secretion and T cell proliferation and function are significantly inhibited by the unique PD-1 expressed on the T cell surface, which eventually leads to immunosuppression [72]. ICP blockade has yielded success in the clinic, and seven ICIs have been received FDA approval since 2011 [66, 73].

## Induction of immunomodulation by chemotherapy

Chemotherapy has been partially successful in treating many types of cancers. Some chemotherapeutic agents kill the tumor cells not only by inducing non-immunogenic apoptosis and cytotoxic effect but also by creating immunological changes and stimulating the host’s immune responses [74]. Several mechanisms involved in immune activation induced by chemotherapy comprise ICD induction, elimination of immunosuppressive cells in the TME, and sensitizing tumor cells to immune attack [75].

### Immunogenic cell death

Killing tumor cells by chemotherapy through induction of ICD or immunogenic apoptosis results in the secretion of damage-associated molecular patterns (DAMPs), like calreticulin (CRT), high mobility group box 1 (HMGB1) and adenosine triphosphate (ATP). Exposure of CRT on dying tumor cell surface leads to antigen engulfment and stimulation of uptake and presentation of tumor-specific antigens by DCs, which in turn causes activation of CTLs to kill tumor cells [76–78]. HMGB1 also induces antigen-specific T cell-mediated immune response by promoting DCs to attach to dying cancer cells [79]. ATP's release facilitates the recruitment and differentiation of T cells and DCs through the autophagy pathway [80]. Some chemotherapeutic agents such as oxaliplatin, idarubicin, epirubicin, cyclophosphamide (CP), and doxorubicin (DOX) can induce ICD [81].

### Elimination of immunosuppressive cells in the TME

Some immunosuppressive cells such as M2-type tumor-associated macrophages (TAMs), regulatory T cells (T_reg_s), and myeloid-derived suppressor cells (MDSCs), which are found in the TME, suppress anti-cancer activity of immune cells. Anti-inflammatory IL-10 and TGF-β released by T_reg_s and TAMs directly suppress T cells' cytotoxic activity and inhibit co-stimulatory ligand expression on DCs, which induces anergy. Moreover, MDSCs inhibit T and NK cells' proliferation and activity by expanding T_reg_s and proliferation to M2-type MQs [82–84]. Chemotherapeutic agents such as cisplatin (CDDP) plus paclitaxel (PTX) directly augment the TME by eliminating these immunosuppressive cells and anti-inflammatory cytokines [85].

### Sensitization of tumor cells to immune attack

CTLs release perforin and granzyme B (Grz B) after interaction with tumor cells. Chemotherapeutic agents can enhance the permeability of tumor cell membrane to Grz B, which leads to enhanced uptake of Grz B and sensitization of tumor cells to CTLs [86].

## Chemotherapy and the overlap between innate and adaptive immunity

**Fig. 2:**
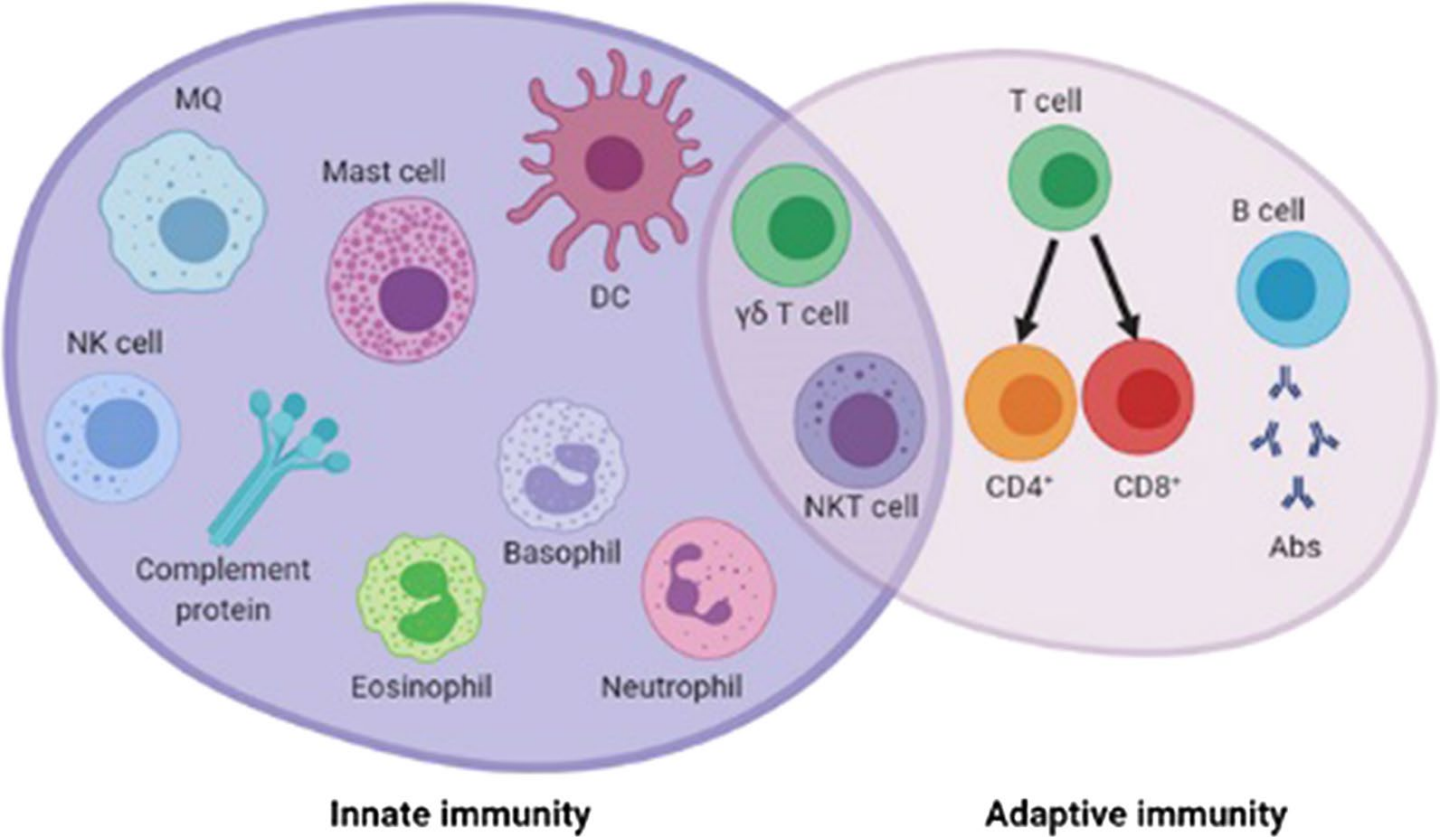
The representational overlap between innate and adaptive immunity

The immune system is divided into two components comprising innate and adaptive immunity, which indicate key roles in creating non-specific and antigen-specific immune responses against cancer, respectively [87]. Moreover, there is an essential interface between these two components, such as natural killer T (NKT) cells and gamma-delta (γδ) T cells that are promising candidates for cancer immunotherapy owing to the modulation ability of both innate and adaptive immunity [88]. The representational overlap between innate and adaptive immunity was illustrated in Fig. 2. γδ T cells recognize different types of tumor cells in an MHC-independent manner and produce PICs such as IFN-γ. Two strategies, comprising adoptive transfer of γδ T cells to patient following ex vivo expansion of them, and in vivo stimulation of γδ T cells using systemic administration of synthetic phosphoantigens (pAgs) or amino-bisphosphonates (N-bis) (pamidronate or zoledronate) have been applied in γδ T cell-based cancer immunotherapy [89, 90]. NKT cells, a subset of CD1d-restricted T cells, have also been proved as a promising boon in manifestation of cancer immunotherapy through a variety of mechanisms, including killing tumor cells directly, influencing immunosuppressive cells in the TME, secretion of cytokines, and stimulating tumor-specific T cells and NK cells which leads to elimination of tumor cells [91, 92].

A combination of γδ T cell- and NKT cell-based therapies with chemotherapy leads to significant cancer treatment efficiency due to their potent anti-tumor cytotoxicity. In fact, these immune cells recognize and kill tumor cells using various mechanisms, and most probably, chemotherapy will enhance their cytotoxicity through one of these pathways [93]. Co-encapsulation of alendronate, a bisphosphonate which activates γδ T cells and DOX, a chemotherapeutic agent in a pegylated liposome, leads to a synergistic anti-tumor effect in breast tumor models [94]. In another study, a high synergistic effect and the highest apoptosis level were achieved when breast cancer cell lines were treated with PTX followed by zoledronic acid compared to reverse sequence or simultaneous treatment. Before immune cell therapy, administration of chemotherapeutic agents could sensitize tumor cells to immunotherapy, while posing no risk of immune cell suppression, which is usually occurred in simultaneous administration. As a result, such combination therapies' synergistic effects appear to be sequential-dependent [95, 96].

## Balancing the pros and cons of chemoimmunotherapy

CIT as the miracle cure is now being utilized through many approved therapies for major cancer types, including lung cancer, breast cancer, and lymphoma and being improved and hoped in endpoints for the phase (III and V) of clinical trials for colon cancer stage III, CRC [96, 97] and pancreatic cancer [98]. Although chemotherapy has been passed the long way as a standard treatment for cancer, however, may not be effective enough as monotherapy in the palliative treatment of patients with cancer due to dose-dependent cytotoxicity. Low-dose chemotherapeutic agents often are ineffective and cause recurrence and metastasis of tumor cells while the administration of high doses leads to severe side effects and immunosuppression; therefore, systemic toxicity will subsequently appear. Moreover, tumor cells have evolved various mechanisms to escape from the immune system because the TME usually plays an immunosuppressive role in multiple types of cancers [99]. Hence, immunotherapeutic approaches will be useful in cancer treatment. Nonetheless, immunotherapy as monotherapy also is not sufficient to overcome tumor cells, due to the attendance of immune system inhibitor cells in the suppressive TME and lack of cytotoxic cells penetration into the tumor. Considering the limitations of chemotherapy and immunotherapy, preclinical and clinical studies aim to enhance the anti-cancer efficacy by combining monotherapies [100, 101]. Treatment with chemotherapy creates necrotic and apoptotic cells in tumor tissue. These materials contain antigens and CTL epitopes which can be released into lymphatic or blood vessels or incorporate into APCs like DCs. In the following, APCs present the derived epitopes and antigens to CTL precursors. As a result, chemotherapy treatment creates an antigen specific immune response and CIT achieves the improvement in the results of both immunotherapy and chemotherapy. In fact, when chemotherapy is combined with immunotherapy, low concentrations of chemotherapeutic drugs is expected to have maximum effect on tumor cells and minimum side effect on normal cells [102, 103].

In general, cancer immunotherapies work through immunomodulating the characteristics of the TME, stimulating the function of T cells, eliminating immunosuppressive immune cells, and finally augmenting endogenous immunity to inhibit tumor growth [17].

One of the promising cancer immunotherapy strategies is the utilization of immunomodulators. The immunomodulation is based on stimulating the function of T cells by blocking or activating regulatory receptors using antibodies, which prevents the progression of cancer. Recently, antibody-based immunotherapy has shifted to targeting immune cells instead of cancer cells. The most critical immunomodulatory antibodies are ICIs that target the PD-1 and CTLA-4 inhibitory receptors on the surface of T cells and, by binding to them, activate anti-tumor T cells to destroy tumor cells [104, 105]. Immunomodulation effects of ICIs may be complemented by the immunogenic effects of chemotherapeutic agents such as increasing mutation burden and neoantigen load, enhancement of T-cell priming and recruitment to the tumor, and increasing MHC I expression to promote antigen presentation [106]. Several randomized clinical trials demonstrated that combining ICIs with chemotherapy may improve their anti-tumor activity in different types of tumors, especially NSCLC [107, 108]. One research group showed that the addition of chemotherapy to PD-1 blockade resulted in improved clinical response in patients with metastatic melanoma [109].

Another modality of cancer immunotherapy is targeting immunosuppressive elements such as MDSCs and T_regS_ in the TME, which demonstrates an essential role in cancer progression and metastasis through inhibition of proliferation and activation of killer T cells. Several immunotherapy approaches are applied to eliminate MDSCs and T_regS_ or impair their immunosuppressive function in different malignancies [110]. Moreover, beyond their direct cytotoxic effects on tumor cells, chemotherapeutic drugs like immunotherapeutic agents can eliminate or inactivate MDSCs and T_regS_ through several mechanisms. Likewise, it has been shown that CP reduces the number of T_regS_ via induction of apoptosis in a mouse model, without affecting T effector viability, which can be attributed to higher proliferation rate of T_regS_ compared to other cells [111]. Furthermore, PTX was reported to reduce the number of MDSCs by inducing differentiation of MDSCs into non-immunosuppressive cell types, DCs [112]. Therefore, the combination of these chemotherapeutic agents with immunotherapy approaches that target MDSCs or T_regS_ appears to be effective in cancer treatment. For instance, the combination of CP and immunotherapy resulted in improved overall survival in colon cancer models [113]. Moreover, it has been reported that inhibition of exosome formation using amiloride blunts the immunosuppressive activity of MDSCs and enhances the anti-tumor efficacy of CP in different mouse tumor models [114].

Immune activation at the forefront of cancer immunotherapy plays a role via several immune stimulants such as cytokines and agonists. Cytokines have several functions, including induction of DC maturation, proliferation and activation of T and NK cells, and enhancement of MHC expression on MQs and DCs [115]. Similarly, some chemotherapy drugs, including CP, have been shown to promote DC maturation [116]. DOX and oxaliplatin also induce T cell stimulation through facilitating tumor antigen uptake by DCs. Due to cytokines and chemotherapy drugs' common effects on immune activation, their combination leads to a synergistic effect in various cancers [117, 118].

**Fig. 3:**
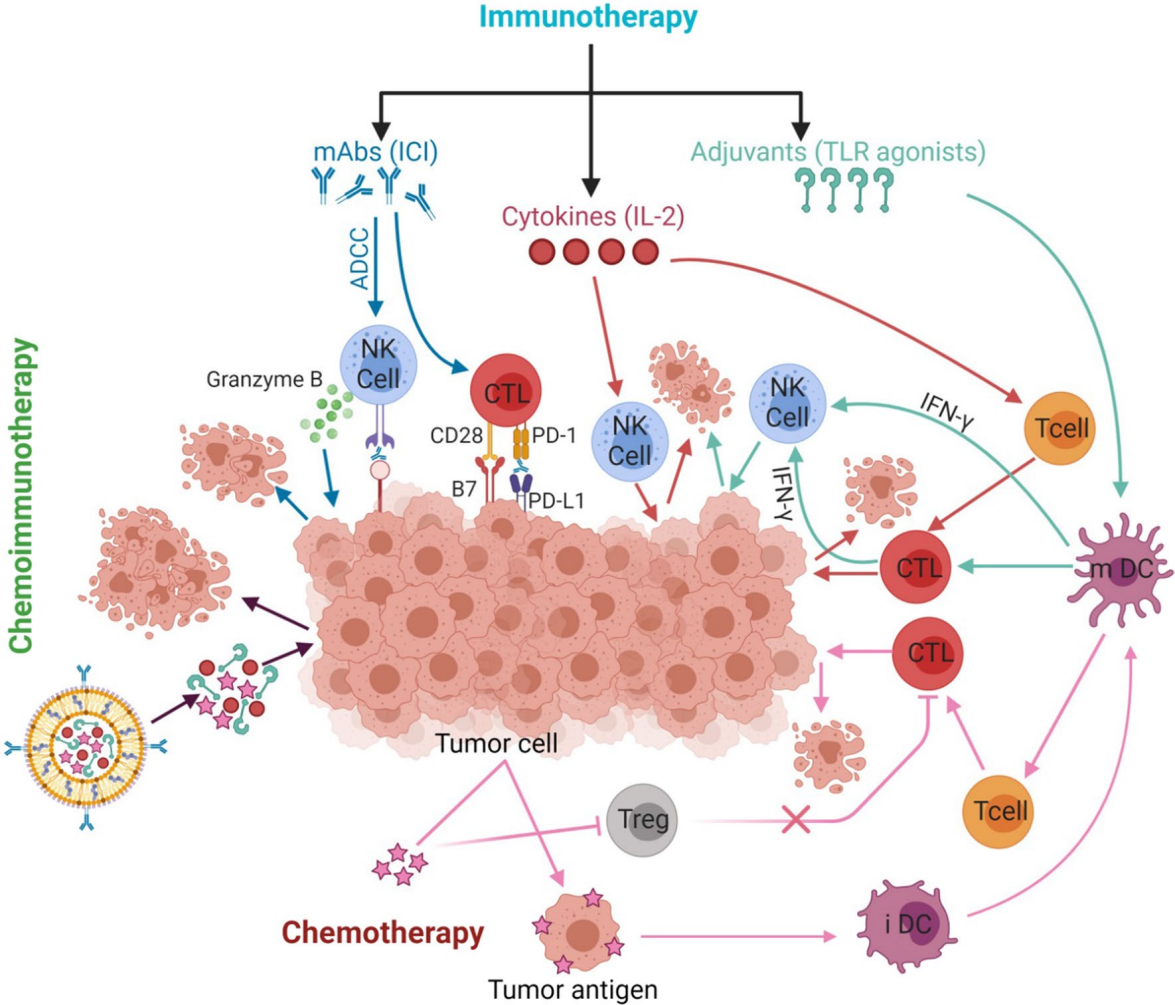
The full gating functional pathway of chemotherapy and immunotherapy and chemoimmunotherapy strategies in tumor-fighting

The full gating functional pathway of chemotherapy and immunotherapy and CIT strategies in tumor-fighting with supporting players in the TME was described in Fig. 3.

### Nanoparticle-mediated chemoimmunotherapy

Despite the promising benefits of CIT in cancer treatment, there are still critical challenges, including the obstacles in the simultaneous delivery of therapeutic agents to target tissues and cells. Chemotherapeutic drugs and immunotherapeutic agents usually have different physicochemical properties and mechanisms and may affect different targets in vivo. They also have distinct PK and in vivo distribution, which results in unpredictable drug ratios at tumor tissues. Furthermore, the unstable structure of most immunotherapeutic agents against enzymatic degradation and chemical conditions leads to the loss of their biological activity [119]. Hence, it is essential to develop a carrier which can load and deliver these two agents simultaneously and achieve synergistic and efficient combination therapy [120].

The manifestation of nanosized carriers has facilitated the rational integration of these distinct approaches and increased the efficacy of CIT to achieve a potential anti-cancer treatment. Harnessing of NPs decreases off-target side effects by increasing therapeutic agents' exposure to target cells and increases the stability of drugs by protecting them from degradation. Moreover, nano-based co-delivery systems could ascertain the targeted delivery and controlled release of drugs, remodel the immunosuppressive TME and ameliorate the in vivo PK behaviors [121, 122]. Accordingly, several types of NPs with different physicochemical properties, including lipid NPs like liposomes, polymeric NPs, metallic or inorganic NPs like mesoporous silica nanoparticles (MSN), hydrogels, cell-derived nanovesicles like exosomes, and hybrid NPs are currently being developed as delivery systems in CIT [123–126]. Cationic lipids and polymers and hybrid NPs have been widely used as the common choice's carriers over the recent decade [127–129]. Metallic or inorganic NPs such as MSN [127, 128], graphene oxide [130], and black phosphorus [131], indicate promising roles including the latest immunogenicity, feasible potential in functionalization and synergized delivery with photothermal therapy (PTT) and photodynamic therapy (PDT) in CIT [132, 133]. Nanogel with the ability to change the core and shell structures leads to responsive functional performance for image cellular tracking and sustained delivery in CIT design [134–136].

The application and manipulation of biomimetic NPs indicate promising role in CIT such as cellular or molecular agents including high-density lipoproteins (HDL), low-density lipoproteins (LDL) [137], albumin [138], and exosome [139], therefore they can emerge predominant potential in CIT perspective. Likewise, some factors, such as biocompatibility, biodegradability, stability in favor of long-term storage, and intended release profile of payloads and delivery of them to target and administration route, should be considered when choosing a good nanocarrier [140, 141].

In studies were surveyed, different immunotherapy agents are used in NP-mediated CIT to create these consequences, which included firstly, cytokines such as IL-2, IFN-γ, IL-7, and IL-15 that are used alone or as a cocktail in various studies to facilitate the growth and activation of different immune cells (DCs, CD4^+^, and CD8^+^ T cells, NK cells, and neutrophils). Carrier-mediated combination of chemotherapy drugs and cytokines was summarized in Table 1. Secondly, immune-stimulating adjuvants like L-Arg, and TLR agonists including TLR4 agonist LPS and detoxified derivatives of LPS like sodium phthalate (SP-LPS) and phthalate (P-LPS) salt of parent LPS, TLR3 agonist poly I:C, TLR7 agonist imiquimod (R837), and TLR9 agonist CpG-ODN which induce an efficient anti-tumor response by activating MQs, inducing effector immune cells such as CD8^+^ T cell and increasing levels of various PICs. Carrier-mediated combination of chemotherapy drugs and immune adjuvants was summarized in Table 2. Thirdly, mAbs such as anti-CD47 and anti-CD326 antibody, and ICIs like PD-L1, PD-1, CTLA-4 mAbs, which are involved in targeted or active delivery of therapeutic agents and may increase specific retention and uptake. Carrier-mediated combination of chemotherapy drugs and mAbs was summarized in Table 3.

**Table 1:** Carrier-mediated combination of chemotherapy drugs and cytokines

Carrier design (Structure/Injection route)	Chemotherapy agent	Cytokines	Tumor type	Synergic actions and advantages of NPs in the combination	Refs.
Polymeric NPs (TMC/SC)	DOX	rhIL-2	Hepatic tumor	Protection of rhIL-2 from enzymolysis without any damage on its bioactivity Considerable inhibition of tumor growth and enhancement of IgG and CTLs levels compared with free drug	[142]
Hybrid NPs (Lipid coated MSNs/IV)		ATRA + IL-2	Melanoma	Enhancement of anti-tumor efficacy and considerable delay in tumor growth and metastasis Activation of TILs and NK cells Induction of IL-12 and IFN-γ secretion and downregulation of MDSCs, IL-10, and TGF-β	[120]
Polymeric NPs (PLGA as core and PEO–PPO–PEO as shell/IV)		IFN-γ		Excellent synergistic anti-tumor efficiency Activation of CD4^+^ T cells, CTLs, and NK cells Induction of IL-2 and TNF-α secretion and downregulation of expression of IL-10 and TGF-β	[143]
Hydrogel NPs (PELG-PEG-PELG)		IL-2 + IFN-γ		Increased anti-tumor efficacy toward free drugs due to sustained release of drugs Increased proliferation of CD3^+^/CD8^+^ and CD3^+^/CD4^+^ T cells	[144]
Cell-derived nanovesicles (DC 2.4/IV)		IL-2, IL-2 + IFN-γ	Melanoma and breast cancer	Efficient inhibition of primary 4T1 tumor progression and lung metastasis of breast cancer Enhancement of DC maturation, promotion of infiltration and activation of CD8^+^ cells and NK cells Increase in recruitment of Ly6G^+^ neutrophils and CD45^+^ immune cells	[145]
Nanogels (PPLG and HPCS/PPLL/SC)		rhIL-2 + rhIFN-γ	NSCLC and breast cancer	Prolonged and continuous release of payloads Significant inhibition of tumor cell proliferation Synergistic anti-cancer efficacy via regulation of apoptosis-related genes in xenograft tumor-bearing mice	[146]
Polymeric NPs (PLGA-mPEG-PLGA/IV)	PTX	IL-2	Melanoma	Remarkable inhibition of tumor growth and metastasis Prolonged overall survival of treated mice in comparison to chemotherapy or cytokine therapy alone Promotion of tumor immunogenicity and the anti-tumor response of immune cells	[147]
Nanogels (Erythrocyte membrane coated nanogels/IV)			Metastatic melanoma (lung metastasis)	Extended in vivo circulation time Increase in anti-tumor activity and improvement in lung metastasis inhibition of PTX/IL-2 loaded nanogel compared to PTX or IL-2 loaded nanogel alone Decrease in number of immune-suppressive cells and enhancement of immune effector cells in the TME	[148]
Polymeric NPs (mPEG-PDLLA as core and pluronic F127 as shell/IV)		IL-12	Breast cancer	Significant accumulation of NPs in tumor cells because of their acid-sensitive property Activation of immune effector cells like T cells and NK cells Modulation of the immunosuppressive TME by inhibiting T_regS_ and inducing differentiation of M1-type MQs Prolonged survival of tumor-bearing mice	[149]
Polymeric hydrogels (mPEG-b-PELG-based hydrogels/SC)	CDDP	IL-15	Melanoma	Inducing cell cycle arrest, synergistic anti-cancer efficacy, and reduced systemic toxicity compared to monotherapy Enhanced anti-tumor immunity owing to suppression of T_regS_ and activation of NK cells and CTLs	[150]

*TMC* *N*,*N*,*N*-trimethyl chitosan, *rhIL-2* recombinant human IL-2, *SC* subcutaneous, *IgG* immunoglobulin G, *ATRA* all-trans retinoic acid, *IV* intravenous, *PLGA* poly(lactic-co-glycolic acid), *PEO* poly(ethylene oxide), *PPO* poly(propylene oxide), *PEG* poly(ethylene glycol), *PDLLA* poly(ethylene glycol)-block-poly(d,l-lactic acid), *PPLG* poly(ethylene glycol)-b-poly(L-glutamic acid), *PELG* poly(ethylglutamate), *HPCS/PPLL* hydroxypropyl chitosan/poly(ethylene glycol)-b-poly(L-lysine)

**Table 2:** Carrier-mediated combination of chemotherapy drugs and immune adjuvants

Carrier design (Structure/injection route)	Chemotherapy agent	Immune adjuvants	Tumor type	Synergic actions and advantages of NPs in the combination	Refs.
Hybrid NPs (G4-Arg^a^/PLGA-PEG-PLGA hydrogel/IV)	DOX	L-Arg	Breast cancer	High therapeutic efficacy and great tumor growth inhibition in 4T1 cells-xenografted mice Synergistic immune therapy through the production of NO by providing a substrate (L-Arg) of iNOS in MQs	[151]
Lipid NPs (TH peptide-modified liposomes/IV)	PTX	αGC + PD-L1 (not encapsulated in NP)	Melanoma and lung metastasis	Upregulation of IFN-γ, maturation of DCs, and activation of NKT cells Significant anti-metastatic effect, enhanced CTL responses, and prolonged survival	[152]
		αGC + acetyl-CoA ACAT-1 inhibitor avasimibe (not encapsulated in NP)		Inhibition of growth and metastasis of melanoma tumors Promotion of the anti-tumor effect via stimulation of CTL responses and formation of TCR Induction of apoptosis through inhibition of ACAT-1 due to an increase in free cholesterol level	[153]
Polymeric NPs (PLGA/Peritumoral)		TLR4 agonist, P-LPS	Melanoma	Increased anti-tumor immune response at the TME compared to PTX and P-LPS alone Activation of APCs and T cells in the tumor site and induction of Th1 immune response Enhancement of TNF-α, IFN-γ, and IL-12 secretion Increase in the infiltration of MQs, DCs, and CD4^+^ and CD8^+^ T lymphocytes	[154]
Polymeric NPs (PLGA/IV)		TLR4 agonist, SP-LPS	Melanoma and MQ model	High amount of PTX in tumor mass compared to commercial PTX followed by IV injection High anti-cancer activity and anti-tumor immune responses Activation of MQs and effector immune cells like cytotoxic T cells and NK cells in splenocytes Secretion of various PICs such as IL-12 and TNF-α	[155]
Hybrid NPs (Conjugate of PTX and SP-LPS/IV)		TLR4 agonist, SP-LPS		Improvement in anti-tumor activity Enhanced percentage of activated immune cells such as MQs, especially M1 type and Th cells Increasing the secretion of IFN-γ, IL-12, and TNF-α	[156]
Polymeric NPs (PEG-PEI/Tail vein)	DOX	TLR2 agonist, Zymosan	Breast cancer	Accumulation of NPs in hypoxic regions of the tumor Inhibition of tumor progression and metastasis, and induction of greater apoptosis Modulation of TAMs differentiation and an increase in expression of Th1 specific cytokines Decrease in VEGFR2 expression and facilitation of anti-angiogenic effect	[157]
Hybrid NPs (MS-Zn micro-rosettes/IV)		Poly I:C sodium salt	Bilateral LLC cell inoculation model	Effective inhibition of tumor growth at the local site Prevention of distant tumor metastases Increased IFN-γ secretion and CD4^+^ and CD8^+^ T cell populations	[127]
Polymeric NPs (PLGA-PEG/IV)		TLR3 agonist, poly I:C + Resiquimod (R848) immune adjuvant + CCL20 chemokine	Lung carcinoma and colon adenocarcinoma	Excellent combination therapeutic efficacy compared to monotherapy Longer survival rate in treated mice Strong activation of specific CTLs in the TME and blood circulation	[158]
Hybrid NPs (Aptamer-G4 PAMAM bioconjugate/IV)		TLR9 agonist, CpG ODN	Prostate cancer	Excellent anti-tumor efficacy and tumor size reduction in mice treated with combination therapy compared to free DOX treated group Higher levels of the IL-1β, IL-12, IL-6, and TNF-α cytokines in MQ cells	[159]
Biomimetic NPs (HDL mimicking/IV)		Aptamer-CpG fused sequences (Apt-CpG-DSPE)	Lung cancer	Enhancement of M1 (MQs) switched the immune-suppressive TME to the immunostimulatory one Facilitation of cell apoptosis and release of tumor-associated antigens Activation of endosomal TLR-9 in infiltrated APCs Enhancement of secretion of PICs such as IL-6 and TNF-α	[160]
Hydrogel NPs (α-Cyclodextrin-PEG/Intratumoral)		CpG NP	Melanoma	Modulation of TME toward immune-suppressive condition Enhancement in the number of CTLs and ratio of CD8^+^ T/T_reg_s Reduction in the number of MDSC and M2-like TAMs	[134]
Polymeric NPs (AC-CS-PpIX micelle and PBA-PEG-PCL (DOX)^b^/Intratumoral and IV)		TLR7 agonist, Imiquimod (R837)	Breast cancer and MQ models	Enhanced anti-tumor immune response Enhanced expression of IL-6, TNF-α, IL-1β, and IFN-γ, and decrease in level of IL-10 expression Higher tumor inhibition rate (85%) and an improved survival rate of treated mice (80%)	[161]
Polymeric NPs (γ-PGA/Intratumoral)	PTX	TLR-7 agonist, Imiquimod	Melanoma, lung cancer, and cervical cancer	Extreme prevention of tumor growth Enhanced activation and proliferation of the DCs and secretion of PICs and Th1 cytokines Enhancement of the population of DCs and MQs in the tumor-draining lymph node	[103]
(Lipophilic prodrugs nanoassemblies/IV)		TLR7 agonist, Imiquimod	Breast cancer	Effective induction of apoptosis and inhibition of tumor growth and angiogenesis, with no tumor recurrence Stimulation of DCs through collaboration of TAAs with R837 leading to potent tumor-specific immune response	[162]
Biomimetic NPs (HDL nanodiscs/Intratumoral)	DTX	TLR9 agonist, CpG ODN	Colon carcinoma	Maximum anti-tumor efficacy and minimum off-target side effects Significant improvement in overall survival in combination-treated mice toward mice treated with DTX alone	[163]

*αGC* α-galactosylceramide, *ACAT-1* acetyltransferase-1, *PEI* polyethyleneimine, *PAMAM* poly amidoamine, *HDL* high density lipoprotein, *γ-PGA* poly(γ-glutamic acid), *AC-CS-PpIX* acetylated-chondroitin sulfate-protoporphyrin IX, *PBA-PEG-PCL* phenylboronic acid-polyethylene glycol-polycaprolactone, *DTX* docetaxel

^a^G4-Arg: fourth-generation L-arginine-rich dendritic NPs; ^b^DOX and TLR7 were delivered using two types of carriers, separately

**Table 3:** Carrier-mediated combination of chemotherapy drugs and mAbs

Carrier design (Structure/injection route)	Chemotherapy agent	mAbs	Tumor type	Synergic actions and advantages of NPs in the combination	Refs.
HDL nanodiscs (ApoA1 mimetic peptide or phospholipids^a^/IV)	DOX	anti-PD-1	Colon adenocarcinoma	Significant regression of colon carcinoma tumors and inhibition of tumor relapse in mice compared to monotherapy or carrier-free dual therapy Induction of long-lasting immunity and delayed tumor growth with no obvious off-target side effects Recruitment of the highest number of CD8α^+^ T cells into the TME and development of systemic antigen-specific CD8α^+^ T cell responses	[164]
Denderimer NPs (G4-PAMAM)		mAb against HER-2, Trastuzumab	Breast cancer	Remarkable cellular uptake, cytotoxic effect, and significant internalization of conjugates to the HER-2 positive cells Synergistic therapeutic effect and enhanced selectivity compared to free drugs and PAMAM-trastuzumab, indicating that DOX dose and thus the cardiotoxicity caused by DOX could be reduced	[165]
Hybrid NPs (Enzyme and pH dual-sensitive micelle-liposome/IV)	PTX	PD-1/PD-L1 inhibitor HY19991		Significant anti-cancer efficacy and high tumor inhibition and lung metastasis suppression rate Increased T cells infiltration in tumor tissues and decrease in cancer stem cell population Prolonged survival time of mice	[166]
Denderimer NPs (G4-PAMAM-PEG/IV)		mAb against HER-2, Trastuzumab		Increased therapeutic efficacy of the conjugate in animal models	[167]
Lipid NPs (pH-sensitive liposomes)	DTX	Anti-PD-L1 blocking antibody	Melanoma	Significant tumor inhibition via high selectivity Activation of tumor-specific CTLs High anti-proliferation efficacy and prolonged survival time	[168]
hydrogel NPs (ROS-responsive hydrogel/Peritumoral)	GEM	Anti-PD-L1 blocking antibody	Melanoma and breast cancer	Induction of an immunogenic tumor phenotype and immune-mediated tumor regression Excellent tumor inhibition and intratumoral infiltration of CD8^+^ and CD4^+^ T cells Reduction of tumor-infiltrating MDSCs	[169]

*ROS* reactive oxygen species, *HER-2* human epidermal growth receptor 2, *ApoA1* apolipoprotein A1, *GEM* gemcitabine

^a^This is not co-encapsulation and only DOX is encapsulated in nanocarrier

In addition to the three categories mentioned above, some other immunotherapy agents have also been studied in combination with chemotherapy drugs using different carriers, which were summarized in Table 4. For example, indoleamine 2,3 dioxygenase (IDO), which is overexpressed enzyme in tumor cells, enables tumors to escape immune surveillance. Likewise, 1-methyltryptophan and indoximod (as IDO inhibitors) combined with chemotherapeutic agents elicited regression of tumors significantly.

**Table 4:** Other combination of chemotherapy drugs and immunotherapy agents

Carrier design (Structure/Injection route)	Chemotherapy agent	Immunotherapy agents	Tumor type	Synergic actions and advantages of NPs in the combination	Refs.
Lipid NPs (Pegylated liposome/IV)	DOX	Alendronate	Female Balb/C and Sabra tumor models	High loading efficiency of DOX and increased stability in biological fluids More potent activation of the inflammasome pathway leading to 40-fold greater secretion of IL-1β High therapeutic efficacy due to synergy of alendronate and DOX	[170]
(Hydrogel/SC)		Melittin-RADA32	Melanoma	Considerable tumor inhibition with the activated NK cells recruitment in the tumors Regulation of innate immune cells, direct anti-cancer and immune-stimulating capabilities Activation of DCs of draining lymph nodes, production of CTLs, and depletion of M2-like TAMs	[171]
(pH-sensitive smart nanocubes/IV)		pOVA vaccine	Melanoma, MQ model and lung carcinoma	Higher anti-tumor efficacy, longer survival rates, and increased tumor inhibition ratio compared to monotherapy Higher OVA protein production Provoking humoral immunity after a single injection and significant humoral immunogenic memory production	[172]
Polymeric NPs (Polymerized β-cyclodextrin/Intratumoral)	PTX	NO	Melanoma, mammary carcinoma, lymphoma and colon carcinoma	Synergistic cytotoxicity and induction of ICD on tumor cells Activation and expansion of DCs leading to expansion of CTLs	[173]
(Dual-pH-sensitive micelle system /Tail vein)		LXR agonist RGX-104	Breast cancer	Significant tumor accumulation, as well as tumor growth suppression Reducing immunosuppressive MDSCs levels and increasing infiltration and anti-tumor effect of CTLs Effective increase in expression of ApoE in tumor tissues Suppression of TGF-β and IL-10 production and enhancement of the number of CD4^+^ and CD8^+^ T cells	[174]
Hybrid NPs (HA coated cationic albumin NPs/Tail vein)	Celastrol	IDO inhibitor, 1-methyltryptophan	Pancreatic cancer	Increase in cytotoxicity, apoptosis induction, and tumor inhibition Downregulation of the immunosuppressive TME through upregulating CD4^+^ T cells in the spleen	[175]
Nanogel (Folated pH-degradable PVA/Tail vein)	DTX	IDO1 inhibitor, NLG919	Breast cancer	Increased intratumoral infiltration of CTLs and NK cells and inhibition of MDSCs infiltration Regulation of IDO1-mediated immunosuppressive TME	[136]
Lipid NPs (Liposome/IV)	DOX	IDO1 inhibitor, Indoximod	Metastatic breast cancer	Significant increase in anti-breast cancer immune response Remarkable tumor cell elimination at the primary tumor sites, as well as metastatic sites Activation of CTLs, depletion of T_reg_s, and enhancement in CD8^+^/FOXP3^+^ T cell ratios Less toxicity in liver, heart, and kidney compared to free DOX	(176)

*HA* hyaluronic acid, *PVA* polyvinyl alcohol, *LXR* liver-X nuclear receptor, *pOVA* plasmid ovalbumin, *NO* nitric oxide

## Conclusion and perspective

The understanding of immunotherapeutic strategies, including using tumor-specific mAbs, ACT, cytokines, and adjuvants in passive strategy and blockade ICP and activation of DC as active approach along with chemotherapy agents could boost CIT. Induction of immunomodulation action through ICD or eradication of immunosuppressive cells in the TME associated with utilizing some of the chemotherapy drugs, which play a CIT role alone or in combination with immunotherapy agents due to unique and effective paradigms can promote the synergistic performance in cancer CIT. Furthermore, the interplay of chemotherapy and the interface between innate and adaptive immunity, such as γδ T cell- and NKT cell-based therapies leads to significant cancer treatment efficiency. Continued advancement in CIT, which is mediated by NPs can improve the treatment of cancers, not to mention the meaningful progress in the development of CIT not only enlighten a possible therapeutic application of anti-cancers but also apprehend a potential for harnessing in interdisciplinary research. Emerging advances in the development of NPs and discover new chemotherapeutic and immunotherapeutic agents, enable the revolution expected for more using of CIT to overcome the hurdles in developing successful new drugs and will make the perspective of CIT for better planning of long-lasting cancer treatment.

## Data Availability

Not applicable.

## Abbreviations

ALL: Acute lymphoblastic leukemia
ATP: Adenosine triphosphate
ACT: Adoptive cell transfer therapy
APCs: Antigen-presenting cells
CRT: Calreticulin
CIT: Chemoimmunotherapy
CAR: Chimeric antigen receptor
CpG-ODN: Cytosine-phosphate-guanosine oligodeoxynucleotides
DAMPs: Damage-associated molecular patterns
DC: Dendritic cell
FDA: Food and Drug Administration
γδ: Gamma-delta
Grz B: Granzyme B
HMGB1: High mobility group box 1
ICP: Immune checkpoint
ICIs: Immune checkpoint inhibitors
ICD: Immunogenic cell death
IFN: Interferon
IL: Interleukin
LPS: Lipopolysaccharide
MQs: Macrophages
MSN: Mesoporous silica nanoparticles
mAbs: Monoclonal antibodies
MDR: Multidrug resistance
MDSCs: Myeloid-derived suppressor cells
NPs: Nanoparticles
NK: Natural killer
NLRs: NOD-like receptors
PRRs: Pathogen recognition receptors
PK: Pharmacokinetics
poly I:C: Polyinosinic-polycytidylic acid
PD-L1: Programmed cell death ligand-1
PD-1: Programmed cell death protein-1
PICs: Pro-inflammatory cytokines
STING: Stimulator of interferon genes
T_reg_s: Regulatory T cells
TCR: Engineered T cell receptor
TIL: Tumor-infiltrating T lymphocyte
TLRs: Toll like receptors
TGF: Transforming growth factor
TME: Tumor microenvironment
TAMs: Tumor-associated macrophages
